# The importance of ribosome production, and the 5S RNP–MDM2 pathway, in health and disease

**DOI:** 10.1042/BST20160106

**Published:** 2016-08-15

**Authors:** Andria Pelava, Claudia Schneider, Nicholas J. Watkins

**Affiliations:** * Institute for Cell and Molecular Biosciences, Newcastle University, Newcastle upon Tyne NE2 4HH, U.K.

**Keywords:** MDM2, nucleolus, p53, ribosome, ribosomal protein, 5S RNP

## Abstract

Ribosomes are abundant, large RNA–protein complexes that are the source of all protein synthesis in the cell. The production of ribosomes is an extremely energetically expensive cellular process that has long been linked to human health and disease. More recently, it has been shown that ribosome biogenesis is intimately linked to multiple cellular signalling pathways and that defects in ribosome production can lead to a wide variety of human diseases. Furthermore, changes in ribosome production in response to nutrient levels in the diet lead to metabolic re-programming of the liver. Reduced or abnormal ribosome production in response to cellular stress or mutations in genes encoding factors critical for ribosome biogenesis causes the activation of the tumour suppressor p53, which leads to re-programming of cellular transcription. The ribosomal assembly intermediate 5S RNP (ribonucleoprotein particle), containing RPL5, RPL11 and the 5S rRNA, accumulates when ribosome biogenesis is blocked. The excess 5S RNP binds to murine double minute 2 (MDM2), the main p53-suppressor in the cell, inhibiting its function and leading to p53 activation. Here, we discuss the involvement of ribosome biogenesis in the homoeostasis of p53 in the cell and in human health and disease.

## Ribosome production

**Figure 1:**
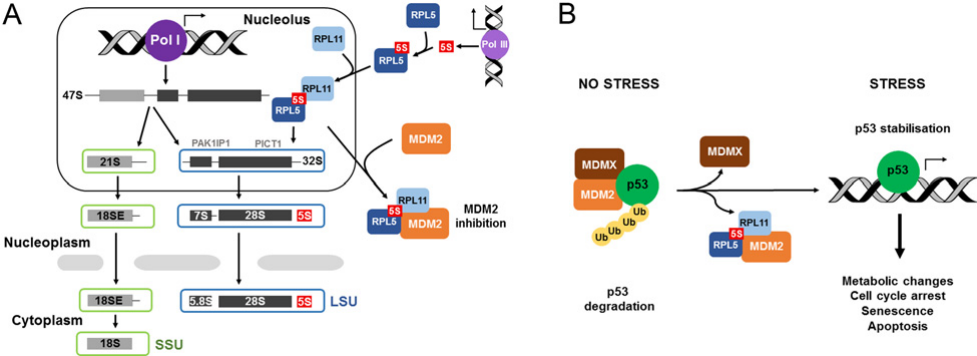
Ribosome biogenesis and p53 signalling (**A**) Schematic representation of ribosome biogenesis and the link between the 5S RNP and MDM2. Three of the rRNAs (18S, 5.8S and 28S) are transcribed by RNA polymerase I (Pol I) in the nucleolus, whereas the 5S rRNA is transcribed by RNA polymerase III (Pol III) in the nucleoplasm. The mature (18S, 5.8S, 28S and 5S) rRNAs and the precursor rRNAs (47S, 21S, 18SE, 32S and 7S) are indicated. The LSU ribosome biogenesis factors PAK1IP1 and PICT1 are shown. (**B**) Illustration of the 5S RNP interaction with MDM2 and the regulation of p53 signalling in both unstressed and stressed cells; Ub: ubiquitin.

The ribosome is a large RNA–protein machine that synthesizes all cellular proteins. Ribosomes consist of two subunits: the small [small ribosomal subunit (SSU); 18S rRNA and 33 ribosomal proteins (RPs)] and large [large ribosomal subunit (LSU); the 28S, 5.8S and 5S rRNAs and 46 RPs] ribosomal subunits [1]. The 28S (25S in yeast), 18S and 5.8S rRNAs are co-transcribed by RNA polymerase I as part of a long single precursor (47S or 35S pre-rRNA in yeast). Ribosomes are assembled on the 47 S precursor transcript as it undergoes multiple processing and modification steps [1] (Figure 1A). RNA polymerase I transcription appears rate-limiting for ribosome production, whereas RPs are produced in significant excess with the unused proteins being rapidly degraded by the proteasome [2]. In contrast, the 5S rRNA is transcribed by RNA polymerase III and is incorporated into the 5S RNP (ribonucleoprotein particle), a ribosome assembly intermediate that also contains the RPs RPL5 and RPL11, before being integrated into the ribosome. Most of the steps of ribosome production occur in the nucleolus, a sub-compartment of the nucleus, with later steps taking place in the nucleoplasm and the cytoplasm [1] (Figure 1A).

Ribosome biogenesis is the major consumer of cellular energy that is up-regulated in cancer, with changes in nucleolar morphology being a hallmark of transformed cells, and down-regulated during cell division and differentiation [3]. Furthermore, ribosome biogenesis is affected by many, if not all, forms of cellular stress including DNA damage, oncogene overexpression (oncogenic stress), hypoxia, oxidative stress and nutritional stress [4]. Not surprisingly, ribosome production is also regulated by oncogenes such as c-*myc* and tumour suppressors such as p53, retinoblastoma protein (Rb) and p14ARF [4]. More recently, it has emerged that ribosome biogenesis also controls multiple cellular signalling pathways and defects in this process can lead to disease.

## The 5S RNP controls the tumour suppressor p53 through MDM2

The tumour suppressor p53 is controlled by the E3 ubiquitin ligase murine double minute 2 (MDM2). Together with the related protein MDMX (also known as MDM4), MDM2 binds to p53, blocking the transcriptional activity of p53 and leading to its ubiquitylation, which targets it for proteosomal degradation [5] (Figure 1B). A number of RPs of both the SSU (RPS3, RPS7, RPS14, RPS15, RPS20, RPS25, RPS26, RPS27, RPS27L and RPS27a) and LSU (RPL4, RPL5, RPL6, RPL11, RPL23, RPL26 and RPL37) has been shown to bind and block the activity of MDM2 [6,7]. The initial model proposed that disruption of ribosome biogenesis leads to the accumulation of free RPs, which bind MDM2 and activate p53 [6]. One problem with this model is that almost all RPs are synthesized in excess and free proteins are rapidly degraded making it difficult to see how they would be stable enough to interact with MDM2 [2]. Moreover, only in the case of RPL5, RPL11, RPL23, RPS3 and RPS27a have the endogenous proteins been shown to bind MDM2 *in vivo* [8–11]. Furthermore, of all the RPs tested, only RPL5, RPL11 and RPS27a have been demonstrated to be essential for p53 activation in response to defects in ribosome biogenesis [8,10,12–14].

Overexpression of the RPs was predicted to result in p53 activation suggesting a role in p53 regulation [6,7]. It was recently shown that RPL4 overexpression activates p53 in an RPL5 and RPL11-dependent manner [7]. One possible explanation for this p53 activation is that overexpression of single RPs could imbalance RP production causing overproduction of the 5S RNP. Although the data on RPL5- and RPL11-mediated regulation of MDM2 are now clear, clarification about which other RPs naturally bind MDM2 and regulate p53 in response to defects in ribosome biogenesis is clearly needed.

RPL5 and RPL11, together with the 5S rRNA, form the 5S RNP, a stable assembly intermediate of the LSU (Figure 1A). Previous data indicate that the 5S RNP, and not the individual RPs RPL5 and RPL11, binds and regulates MDM2 in unstressed cells [8,12,14]. This interaction is enhanced after defects in ribosome production when the 5S RNP accumulates [14]. All three components of the 5S RNP contact MDM2 [14–18] and all three are needed for p53 activation in response to defects in ribosome biogenesis [12,14]. The 5S RNP–MDM2 pathway is needed for activation of p53 by chemotherapeutic drugs (e.g. actinomycin D and 5-fluorouracil), low nucleotide levels in the cell, nutrient starvation and overexpression of the tumour suppressor p14ARF [12,14,19–21]. Ribosome biogenesis and the 5S RNP–MDM2 pathway are also thought to be important for the unfolded protein response [22] and likely to be involved in the response to many forms of stress including hypoxia and oxidative stress. Indeed, only p53 activation by DNA damage has been shown to occur independently of the 5S RNP–MDM2 interaction [23], indicating that the 5S RNP–MDM2 pathway is a major p53 regulatory mechanism in the cell.

The cell contains a large amount of the RPL5/5S rRNA complex, an intermediate in 5S RNP formation (Figure 1A), and the recruitment of RPL11 to the RPL5/5S rRNA complex to form the 5S RNP is rate-limiting [14]. Since individual, unbound RPs are unstable, the 5S RNP complex provides a means by which RPL5 and RPL11 can accumulate when ribosome production is blocked [8,14]. Monitoring the levels of the 5S RNP, which is only present at low levels normally in the cell, therefore provides a good measure of the rate and integrity of ribosome production especially since the mature subunits are very long-lived with estimated half-lives of 5 days in rat livers [24]. Unexpectedly, defects in SSU production also lead to 5S RNP-mediated activation of p53 in the absence of noticeable changes in LSU production. In this instance, reduction in SSU levels is predicted to lead to the up-regulation of RP production and the increased production of the 5S RNP [13].

**Figure 2:**
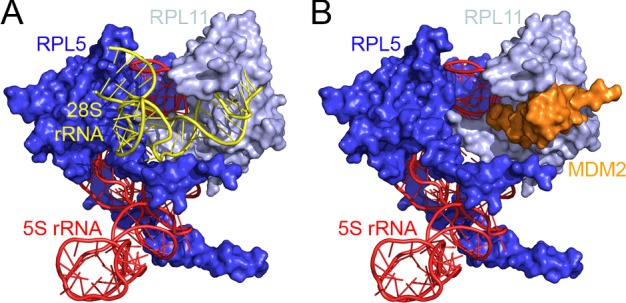
The interaction of MDM2 and the ribosome with the 5S RNP is mutually exclusive Structure of the human 5S RNP bound to (**A**) nucleotides 4237–4288 of the 28S rRNA [51] and (**B**) a fragment of MDM2 including the zinc finger [25]. Surface and cartoon views of the RPs and rRNAs respectively, are shown. Images were generated using PyMOL software.

Recent structural work has shown that the binding of the 5S RNP to MDM2 and to the ribosome are mutually exclusive events [25]. The same binding site in RPL11 is used in both cases (Figure 2) thus preventing the interaction of the ribosome-bound 5S RNP with MDM2. A number of proteins have been linked to the regulation of p53 by the 5S RNP and ribosome production, including the essential ribosome biogenesis factors PAK1IP1 (Mak11 in yeast) and PICT1 (also known as GLTSCR2 and Nop53 in yeast), which are essential for the production of the LSU (Figure 1A) [14,26,27]. These proteins, like all ribosome biogenesis factors, are involved in p53 regulation by performing their basic function in ribosome production.

MDM2 regulates a number of signalling factors in the cell including the p53-related proteins p63 and p73 [28]. It is therefore likely that the 5S RNP regulates multiple cellular signalling pathways through its interaction with MDM2. Recently, RPL5 and RPL11, presumably as part of the 5S RNP, were shown to regulate p73 [29]. However, instead of binding to, and blocking MDM2 activity, RPL5 and RPL11 directly bind to p73 and block the interaction of p73 with the suppressor MDM2. Furthermore, RPL11 has also been shown to bind and suppress the transcriptional activity of the proto-oncogene c-*myc* in response to defects in ribosome biogenesis [30]. In addition, RPL5 and RPL11 co-operatively recruit the RNA-induced silencing complex to degrade the c-*myc* mRNA [30]. It is likely that both the regulation of c-*myc* activity and c-*myc* mRNA stability are regulated by the 5S RNP. It will be interesting to see what other signalling pathways are regulated through the 5S RNP complex.

## The importance of ribosome biogenesis in human health and disease

Changes in ribosome biogenesis have been linked to a wide range of conditions including cardiovascular, neurodegenerative and skeletal disorders as well as cancer [31–34]. An ever-growing number of rare genetic diseases, termed ribosomopathies, have been attributed to defects in ribosome production [35,36]. These diseases arise due to mutations in genes encoding RPs or proteins important for ribosome production. Common clinical manifestations include macrocytic anaemia, skeletal defects, skin problems and, notably, a pre-disposition to cancer. In many cases, p53 levels have been shown to be de-regulated and, in some animal models, the clinical symptoms of the diseases have been shown to be p53-dependent. Evidence suggests that defects in ribosome production in these cases lead to 5S RNP accumulation and subsequent p53 activation. The most-studied ribosomopathies are Diamond–Blackfan anaemia (DBA), Shwachman–Diamond syndrome (SDS), 5q syndrome and Treacher Collins syndrome (TCS). Where the mutant gene has been identified, the majority of DBA patients have mutations in genes encoding RPs including RPS19, RPS24, RPS17, RPS7, RPL35A, RPL5 and RPL11, and defects in pre-rRNA processing are commonly used as a diagnostic tool for this disease [37]. DBA patients present with hypoplastic macrocytic anaemia together with heterogeneous anomalies including skeletal, urogenital and cardiac defects. In DBA animal models some, but not all of the symptoms have been shown to be p53-dependent and the anaemia is dependent on the interaction between the 5S RNP and MDM2 [38]. Like DBA, 5q syndrome presents with macrocytic anaemia and is a form of myelodysplastic syndrome (MDS) [39]. 5q syndrome is caused by deletion of chromosome 5q, which contains the gene encoding RPS14. The anaemia in 5q syndrome has been attributed to haploinsufficiency of RPS14 and is p53-dependent in mouse models [40]. SDS is caused by mutations in the *SBDS* gene, which encodes a protein important for the late, cytoplasmic stages of LSU maturation [41]. SDS patients present with endocrine pancreatic insufficiency, ineffective haematopoiesis and, occasionally, anaemia and skeletal defects. Mutations in TCOF1, and less commonly the RNA polymerase I and III subunits POLR1C and POLR1D, cause TCS. TCOF1 is important for both the transcription and 2′-*O*-methylation of the rRNA [34]. TCS presents with craniofacial anomalies due to proliferation defects of neural crest cells during early development. A mouse model successfully replicated the clinical phenotype by mutating the TCOF1 gene, and further work revealed the craniofacial defects to be p53-dependent [42].

For some time now, ribosome biogenesis has been known to be linked to cancer. Changes in nucleolar structure (as seen by Ag NOR staining) and an increase in ribosome production are two hallmarks of cellular transformation [4]. Strikingly, many anti-cancer chemotherapeutics, including actinomycin D and 5-fluorouracil, block ribosome biogenesis [43] making it a promising target for new cancer therapies [44]. The tumour suppressor p14^ARF^, which needs the 5S RNP for its full capability to activate p53 [14], is also a suppressor of ribosome production [45]. However, the most striking link is seen with the highly abundant nucleolar protein nucleophosmin (NPM1), which is mutated in approximately 30% of leukaemias [46]. More recently, exome sequencing has revealed mutations in RPL5, RPL10, RPL11, RPL22, RPS15 and RPS20 in a variety of cancers but most notably leukaemias [47]. In particular, RPS15 mutations are associated with an aggressive, relapsed form of chronic lymphocytic leukaemia (CLL) and frequently found associated with mutations in the p53 gene [48]. In many ribosomopathies, the patients are pre-disposed to multiple forms of cancer. For example DBA patients have a 5-fold higher risk of cancer than the general population with a 28- to 36-fold higher incidence of acute myeloid leukaemia (AML), osteosarcoma or colon cancer [47]. AML is the most common form of cancer associated with ribosomopathy patients. Solid tumours, such as head and neck tumours or osteosarcomas, are less common but also seen [49]. The increase in cancer rate in ribosomopathy patients appears counter-intuitive since they often have higher than normal levels of p53. However, it could be that the patient's cells become desensitized to p53 or that there is pressure to mutate or affect the p53 pathway when p53 levels are elevated.

Recent work has indicated a role for p53 in the regulation of cellular metabolism. This was highlighted in a mouse model carrying a cancer derived point mutation, C305F, in MDM2 [20]. This mutation blocks the 5S RNP–MDM2 interaction and renders the mouse cells insensitive to p53 activation when ribosome biogenesis is blocked using low levels of actinomycin D [23]. In addition, this mutation also increases the rate the mice develop c-*myc*-induced lymphomas. Interestingly, the MDM2–C305F mutation promotes fat accumulation in mice under normal conditions and hepatosteatosis under fasting conditions [20]. It was further shown that ribosome biogenesis, and the 5S RNP–MDM2 pathway, is the sensor responsible for p53-mediated remodelling of lipid homoeostasis in the liver in response to restricted nutrients. In a separate study, it was found that the protein nucleomethylin (NML) functions to repress rRNA transcription in the livers of mice fed a high-fat diet [50]. NML-null mice showed a reduced lipid accumulation in the liver and these mice did not become obese on a sustained high-fat diet. It is unclear whether NML-mediated suppression of rRNA transcription functions to control liver metabolism through p53, although it is likely that the livers in mice fed with a high-fat diet contain elevated levels of p53. It is therefore clear that the regulation of ribosome production is a major nutrient sensor and critical for the correct response to changes in diet that affect liver function.

## Concluding remarks

Research in the last few years has revealed how intimately ribosome biogenesis is linked to cellular signalling and, in particular, to the control of the tumour suppressor p53. It is now clear that cellular signalling depends on the levels of a ribosome assembly intermediate, the 5S RNP, which together with MDM2, plays a pivotal role in this pathway. This is linked to the basic tumour suppressor/anti-proliferation aspect of p53 and high levels of the 5S RNP cause p53-dependent cell cycle arrest, apoptosis or senescence. However, intriguingly, the 5S RNP/MDM2 pathway also appears to regulate lipid metabolism in the liver in response to changes in diet. Furthermore, the 5S RNP is also linked to the control of other signalling factors such as c-*myc* and p73. Indeed, it is likely that, in the coming years, the 5S RNP will be revealed to be a central hub regulating multiple aspects of cellular signalling.

## Abbreviations

AML: acute myeloid leukaemia
DBA: Diamond–Blackfan anaemia
LSU: large ribosomal subunit
MDM2: murine double minute 2
NML: nucleomethylin
RNP: ribonucleoprotein particle
RP: ribosomal protein
RPL: ribosomal protein of the large subunit
RPS: ribosomal protein of the small subunit
SDS: Shwachman–Diamond syndrome
SSU: small ribosomal subunit
TCS: Treacher Collins syndrome
